# The Virulence Factor Macrophage Infectivity Potentiator (Mip) Influences Branched-Chain Amino Acid Metabolism and Pathogenicity of *Legionella pneumophila*

**DOI:** 10.3390/metabo13070834

**Published:** 2023-07-11

**Authors:** Fabian Nikolka, Mustafa Safa Karagöz, Mohamed Zakaria Nassef, Karsten Hiller, Michael Steinert, Thekla Cordes

**Affiliations:** 1 Department of Bioinformatics and Biochemistry, Braunschweig Integrated Centre of Systems Biology (BRICS), Technische Universität Braunschweig, 38106 Braunschweig, Germany; 2 Institut für Mikrobiologie, Braunschweig Integrated Centre of Systems Biology (BRICS), Technische Universität Braunschweig, 38106 Braunschweig, Germany; 3 Research Group Cellular Metabolism in Infection, Helmholtz Centre for Infection Research, 38124 Braunschweig, Germany

**Keywords:** *Legionella pneumophila*, infection model, metabolism, tracing, mass spectrometry, branched-chain amino acid, macrophage, macrophage infectivity potentiator (Mip), virulence factor, mass spectrometry

## Abstract

*Legionella pneumophila* (*Lp*) is a common etiological agent of bacterial pneumonia that causes Legionnaires’ disease (LD). The bacterial membrane-associated virulence factor macrophage infectivity potentiator (Mip) exhibits peptidyl-prolyl-*cis/trans*-isomerase (PPIase) activity and contributes to the intra- and extracellular pathogenicity of *Lp*. Though Mip influences disease outcome, little is known about the metabolic consequences of altered Mip activity during infections. Here, we established a metabolic workflow and applied mass spectrometry approaches to decipher how Mip activity influences metabolism and pathogenicity. Impaired Mip activity in genetically engineered *Lp* strains decreases intracellular replication in cellular infection assays, confirming the contribution of Mip for *Lp* pathogenicity. We observed that genetic and chemical alteration of Mip using the PPIase inhibitors rapamycin and FK506 induces metabolic reprogramming in *Lp*, specifically branched-chain amino acid (BCAA) metabolism. Rapamycin also inhibits PPIase activity of mammalian FK506 binding proteins, and we observed that rapamycin induces a distinct metabolic signature in human macrophages compared to bacteria, suggesting potential involvement of Mip in normal bacteria and in infection. Our metabolic studies link Mip to alterations in BCAA metabolism and may help to decipher novel disease mechanisms associated with LD.

## 1. Introduction

Legionnaires’ disease (LD) is a severe form of pneumonia caused by the bacterium *Legionella pneumophila* (*Lp*). The bacterium is characterized by various virulence factors that enable it to survive and multiply within host cells. One such virulence factor is the bacterial membrane-associated virulence factor macrophage infectivity potentiator (Mip) which exhibits peptidyl-prolyl-*cis/trans*-isomerase (PPIase) activity. Mip contributes to the intracellular pathogenicity in macrophages and extracellular tissue dissemination of *Lp* [1]. However, how the surface protein Mip helps the bacterium to evade the host immune system is not well understood.

To protect the body against pathogens, immune cells must detect and respond to signals from their surrounding environment. Macrophages undergo significant changes in their metabolism that are crucial for the host’s defense against bacterial infections. Intracellular metabolites, such as mitochondrial TCA cycle-related small molecules, emerge as metabolic regulators influencing immune cell metabolism and function [2,3,4]. Further, nutrient availability, including amino acids and lactate, induces metabolic reprogramming, affecting cellular processes critical for immune responses [5,6].

While much research has focused on the metabolic and molecular changes that occur in host cells during infection, limited information is available on the metabolic strategies employed by *Lp* to evade host immune responses and establish intracellular replication and infection [7]. Amino acids, carbohydrates, and glycerol are essential nutrients for *Lp’s* growth and infection processes [8]. For instance, serine is a critical carbon source for *Lp,* and mutations in genes involved in serine biosynthesis alter bacterial growth [9], indicating the critical role of amino acid metabolism in modulating the outcome of *Lp* infection. *Lp* subverts the host defense and clearance. These defense strategies have evolved over millions of years, leading to the emergence of highly adapted bacterial strains. As reported in earlier studies, Mip modulates the pathogenicity of *Lp* [10] via its effects on bacterial flagellation [11], extracellular matrix (ECM) degradation [12], and apicobasal transmigration in tissues [11]. Thus, Mip may affect cell function and metabolism during infections. While mass spectrometry is commonly used to study the link between metabolism and immunity in mammalian cells [13], metabolic approaches to identify the metabolic consequences of virulence factors in *Lp* are limited.

Here, we explore the concept that the virulence factor Mip may influence metabolism to alter infections. We applied mass spectrometry approaches and established a metabolic workflow to decipher the metabolic impact of altered Mip activity on *Lp* virulence. We confirmed that impaired Mip activity altered bacterial pathogenicity in infections with human macrophages. Our non-targeted mass spectrometry approach identified increased branched-chain amino acid (BCAA) levels in genetically engineered bacteria lacking Mip activity or treated with the PPIase inhibitor rapamycin and FK506. In contrast, rapamycin induced a distinct metabolic phenotype in human macrophage cells, suggesting a potential role of PPIase activity in host cells. Our data indicate that the virulence factor Mip alters bacterial BCAA levels, which may further influence the metabolic homeostasis in infections. Thus, BCAA metabolism might be a potential metabolic vulnerability that can be targeted therapeutically to buffer the outcome of LD.

## 2. Materials and Methods

### 2.1. Cell Culture

The human acute monocytic leukemia THP-1 cell line was procured from DSMZ, ACC 16, and screened negative for mycoplasma contamination. The cells were maintained in RPMI medium supplemented with 10% FBS and cultured in a humidified cell culture incubator at 37 °C and 5% CO_2_. THP-1 monocyte-like cells were differentiated into macrophages using 100 nM PMA for 48 h. To perform the experiments, 1 mL of >80% confluent monocytes were seeded in 12-well plates and differentiated for 48 h at a density of 10^6^ cells per mL. The cells were treated with 25 µM rapamycin for 24 h or 1% DMSO as a negative control after determining the cytotoxicity of rapamycin as 50 µM, following ISO 10993-5:2009 guidelines.

### 2.2. Bacterial Cultures

This study used the *Lp* Corby wild-type [14] and verified *Lp* Corby Δ*mip* bacterial strains [15]. The strains were cultivated on BCYE agar (10 g ACES, 10 g yeast extract, 0.4 g L-cysteine, 0.25 g iron (III) nitrate, and 15 g agar in 1000 mL ddH_2_O) or YEB medium (10 g ACES, 10 g yeast extract, 0.4 g L-cysteine, 0.25 g iron (III) pyrophosphate in 1000 mL ddH_2_O) and maintained at 37 °C. BCYE agar plates were incubated for 72 h, whereas YEB cultures were grown at 37 °C and 200 rpm for 24 h. Kanamycin at a concentration of 25 µg/mL was supplemented in the BCYE agar, solely applied to the Δ*mip* strain.

The growth curves of the bacteria were generated by inoculating the grown cultures into YEB medium, followed by incubating the precultures for 24 h. Subsequently, the grown precultures were adjusted to OD_600nm_ = 0.01 and grown in 250 mL of YEB medium. The OD_600nm_ of the bacterial cultures was measured at regular intervals using Biochrom Libra S22. Six independent biological replicates were conducted for each bacterial strain.

### 2.3. Minimal Inhibitory Concentration (MIC) Assay

The *Lp* Corby strains were cultured on BCYE agar supplemented with or without antibiotics until they attained the stationary growth phase. Subsequently, bacteria were harvested with OD_600nm_ = 0.01 and subjected to a 24 h growth period in a volume of 100 µL YEB medium containing FK506 (CAS #104987-11-3—Calbiochem, dissolved in DMSO) or rapamycin (CAS #53123-88-9—Calbiochem, dissolved in DMSO) in concentrations from 3.125 µM to 50 µM. To assess the effects of the substances, 1% DMSO was used as a control condition. The bacterial growth was monitored by measuring the OD_600nm_ after 24 h with a microplate fluorometer (TECAN Infinite^®^ M Nano, Tecan Group, Maennedorf, Switzerland) with bacteria and without any treatment serving as a negative control to check for contamination. Finally, the values obtained from the plate reader were analyzed using GraphPad Prism software to determine the effects of the substances on the growth of the bacteria from six independent biological repeats.

### 2.4. Infection Experiments

*Lp* strains were cultured on BCYE agar and prepared to a concentration of 10^6^ cells/mL. Macrophage-like THP-1 cells, differentiated for 48 h with 100 nM PMA, were then infected with 100 µL of the bacterial suspension in RPMI + 10% FBS medium at a multiplicity of infection (MOI) of 1:1. The cells were incubated for 3 h at 37 °C and 5% CO_2_, followed by three washes with prewarmed PBS to remove extracellular bacteria. To monitor the uptake of bacteria, cell lysis was performed with 0.1% (*v*/*v*) Triton X-100, and serial dilutions were plated on BCYE agar plates. The replication of intracellular bacteria was assessed by repeating the plating procedure at 24 and 48 h. The number of colony-forming units (cfu)/mL was calculated by counting the plates after incubation for 3–4 days at 37 °C. This process was repeated with six independent, biological replicates (with three cultural replicates each).

### 2.5. Gas Chromatograph–Mass Spectrometry (GC/MS), Sample Preparation, and Analysis

The *Lp* Corby strains were cultured on BCYE until they reached the stationary growth phase. Precultures were inoculated with OD_600nm_ = 0.01 of bacteria in YEB medium supplemented with either 20 µM FK506, 20 µM rapamycin or 1% DMSO, or they were left untreated, using different strains of bacteria. Metabolite extraction was performed with 10^8^ bacteria, which were harvested after 24 h of growth by centrifugation at 4000× *g* for 20 min at 4 °C. The pellets were washed three times with saline solution, centrifuged again, and quenched with 0.25 mL in −20 °C methanol. After adding 0.25 mL of 4 °C cold water, cells were collected in tubes containing 0.25 mL −20 °C chloroform. The extracts were vortexed for 20 min at 1200 rpm and 4 °C and centrifuged at 17,000× *g* for 5 min at 4 °C. The upper aqueous phase was evaporated under a vacuum at 4 °C.

Metabolites from the THP-1 cell culture experiments were extracted, analyzed, and quantified, as previously described in detail [16]. Briefly, the cells were washed with saline solution and quenched with 0.25 mL of −20 °C methanol. After adding 0.25 mL of 4 °C cold water, the cells were collected in tubes containing 0.25 mL of −20 °C chloroform. The extracts were vortexed for 20 min at 4 °C and centrifuged at 17,000× *g* for 5 min at 4 °C. The upper aqueous phase was evaporated under a vacuum at 4 °C.

Derivatization for polar metabolites was performed using a Gerstel MPS with 15 μL of 2% (*w*/*v*) methoxyamine hydrochloride (Thermo Scientific, Waltham, MA, USA) in pyridine and 15 μL of N-methyl-N-(trimethylsilyl)trifluoracetamid (MSTFA) or 15 μL N-tertbutyldimethylsilyl-N-methyltrifluoroacetamide (MTBSTFA) with 1% tert-butyldimethylchlorosilane (Regis Technologies). Derivatives were analyzed by GC/MS using a 30 m DB-35MS + 5 m Duraguard capillary column (0.25 mm inner diameter, 0.25 µm film thickness) installed in an Agilent 7890B gas chromatograph (GC) interfaced with an Agilent 5977A mass spectrometer (MS) operating under electron impact ionization at 70 eV. The MS source was held at 230 °C and the quadrupole at 150 °C, and helium was used as a carrier gas. The GC oven was held at 80 °C for 6 min, increased to 300 °C at 6 °C/min and held for 10 min, and held at 325 °C for 4 min for the MSTFA method, or it was held at 100 °C for 2 min, increased to 300 °C at 10 °C/min and held for 4 min, and held at 325 °C for 3 min for the MTBSTFA method.

Chromatograms were analyzed with MetaboliteDetector software [17]. Peaks were identified with an in-house database consisting of molecular masses, retention indices, retention times, spectra, sum formulas of the compounds, and the ions used for quantifications. Furthermore, quality control measurements and standards were used to account for retention time divergences compared to library measurements. Batch quantification was calculated via group-based compound matching with low retention index differences, high similarity, and compound reproducibility and was normalized to internal standards.

### 2.6. Isotopic Tracing

For tracing experiments, cells were cultured in SILAC RPMI 1640 medium. [^12^C]glucose was replaced with 11 mM [U^13^-C_6_]glucose (Cambridge Isotopes Inc., Tewksbury, MA, USA). All media were supplemented with 10% FBS and adjusted to pH = 7.3. Metabolites were extracted and measured using GC/MS as indicated above. Mass isotopomer distributions and total metabolite abundances were computed by integrating mass fragments using MetaboliteDetector software with corrections for natural isotope abundances, as described previously [17]. Labeling is depicted as mole percent enrichment (MPE). Details on specific fragments have been provided elsewhere [16].

### 2.7. Statistics

Metabolites for heatmaps were tested for significance via ANOVA using a cutoff score of 0.05. Heatmaps depict identifiable and significantly affected metabolites and were visualized using MetaboAnalyst software, version 5.0. GraphPad Prism software, version 9, was used for barplot visualization and statistical analysis. The type and number of replicates and the statistical tests used are described in each figure legend. Data are presented as heatmaps, the mean ± s.e.m, or boxes (25th to 75th percentile with median line) and whiskers (min. to max. values), as described in the figure legends. Main products (MPs) and by-products (BPs) of metabolites were indicated. Tissue culture was conducted in 12-well tissue culture plates with three cellular replicates. *Legionella* experiments were conducted with three replicates. All data are depicted from one representative experiment, and each experiment was independently repeated three times. *p* values were calculated using Student’s two-tailed *t*-test, one-way ANOVA, or two-way ANOVA, and * *p* < 0.05; ** *p* < 0.01; *** *p* < 0.001, and # *p* < 0.0001 as indicated in the figure legends.

## 3. Results

**Figure 1:**
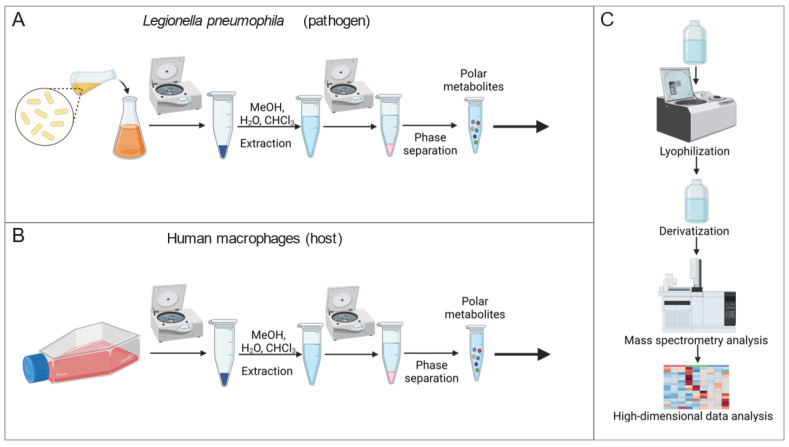
Workflow to quantify metabolism of *Lp* and THP-1 macrophage-like host cells. (**A**) Extraction workflow for *Lp* cultures. (**B**) Extraction workflow for human macrophages. (**C**) Quantification of metabolites with a mass spectrometry-based analytical setup, followed by high-dimensional data analysis and graphic output to visualize metabolic alteration. (Created with BioRender.com, accessed on 26 April 2023).

Mass spectrometry is a powerful approach for quantifying the alteration of metabolite levels in living cells. To identify the metabolic consequences of altered Mip activity, we established a metabolic workflow for reliable quantification of metabolic alterations in *Lp* (Figure 1A). We applied a modified extraction method by Bligh and Dyer [18] and extracted intracellular metabolites of *Lp* by adding an extraction fluid mixture consisting of water, methanol, and chloroform to the bacteria. We quantified metabolism in a non-targeted manner by applying gas chromatography coupled to mass spectrometry (GC/MS). Our established metabolic workflow is a robust and reliable method to analyze intracellular metabolite levels in *Lp*.

Further, we quantified metabolic reprogramming in human macrophages in response to the chemical PPIase inhibitor rapamycin using GC/MS (Figure 1B). To establish a macrophage-like phenotype, we differentiated the human monocyte-like cell line THP-1 using phorbol 12-myristate 13-acetate (PMA). We then applied our established workflow for mammalian cells to extract and quantify the metabolism [16]. High-dimensional data analyses were performed using the MetaboliteDetector computational algorithm (Figure 1C) [17]. Therefore, our established metabolic workflow is critical to identify metabolic signatures in response to altered Mip activity.

### 3.1. Impaired PPIase Activity Influences Lp Growth and Virulence

**Figure 2:**
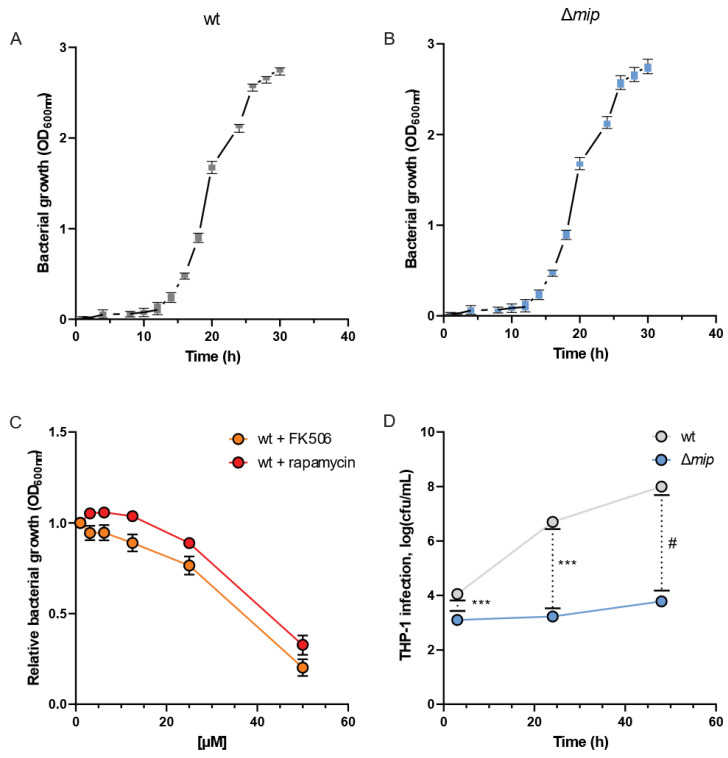
Loss of Mip activity has minor impact on the growth rate but influences infectivity in *Lp*. Growth curves of *Lp* (**A**) wild-type strain and (**B**) Δ*mip* strain. (**C**) The Mip inhibitors FK506 and rapamycin reduce growth at concentrations greater than 35 µM. (**D**) Viable cell numbers after infection of differentiated macrophage-like THP-1 cells are depicted as cfu/mL. Data are presented as mean ± s.e.m or boxes (25th to 75th percentile with median line) and whiskers (min. to max. values) calculated from n = 6 replicates. Changes between wt and Δ*mip* strains were compared using Dunnet’s multiple comparison test with six independent replicates. Statistical significance is indicated by *** *p* < 0.001, # *p* < 0.0001.

To analyze how Mip influences *Lp* infections, we quantified the growth dynamics of the bacteria in the presence and absence of the virulence factor Mip [19]. We assessed the bacterial growth using optical density (OD) measurements at 600 nm at different time intervals and observed clear patterns of log and lag phases of bacterial growth for wild-type (wt) and genetically engineered *mip* knockout (Δ*mip*) strains (Figure 2A,B). Notably, the growth of *Lp* was not influenced by altered Mip activity (see Figure S1 for raw data). This observation is consistent with previous reports [1], further supporting that Mip activity has a negligible impact on bacterial growth.

FK506 (tacrolimus) and rapamycin (sirolimus) are FDA-approved immunosuppressants that target and inhibit the C-terminal peptidyl-prolyl cis-trans isomerase (PPIase) domain of Mip [1]. Therefore, we utilized FK506 and rapamycin as chemical PPIase inhibitors to influence Mip activity and compared the results with genetically modified Δ*mip* strains. We exposed *Lp* to increasing concentrations of the inhibitors and quantified bacterial density after 24 h (Figure 2C). We observed that the lethal dose of the inhibitors for *Lp* growth was approximately 35 µM and in the range of previously reported studies (Figure 2C) [20,21].

To elucidate the significance of Mip activity in bacterial infection, we conducted an infection assay using human macrophage-like THP-1 cells and quantified bacterial entry and replication via colony-forming units. Our genetically modified *Lp* strains lacking Mip activity (Δ*mip*) exhibited a marked reduction in cellular infection of human macrophages compared to wild-type strains (wt) (Figure 2D). Our data align with previous studies [10], confirming the contribution of Mip to bacterial infection.

### 3.2. Metabolic Impact of Genetically Altered Mip Activity in Engineered Lp

**Figure 3:**
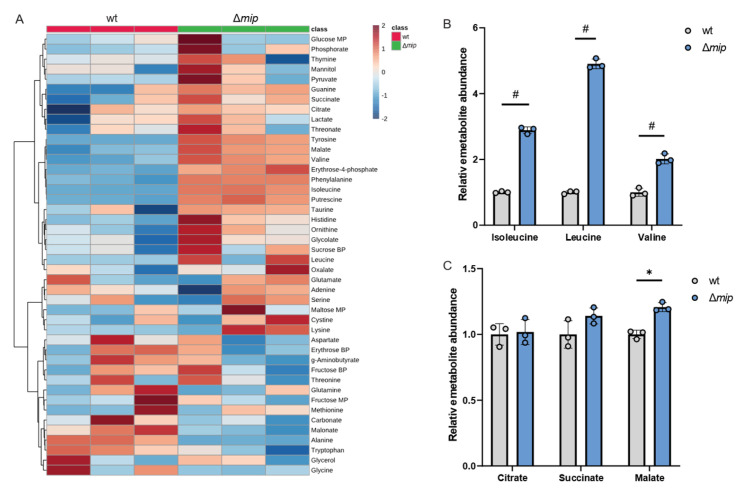
Genetically modified Mip activity (Δ*mip*) influences BCAA metabolism in *Lp*. (**A**) Heatmap depicting untargeted metabolic analysis of wild-type *Lp* compared to Δ*mip* strain. (**B**) Relative metabolite abundance of BCAAs in wild type compared to Δ*mip* strain. (**C**) Relative metabolite abundance of TCA metabolites in wild type compared to Δ*mip* strain. All data are presented as mean ± s.e.m. calculated from n = 3 cultural replicates, and each experiment was repeated independently three times. Relative abundances were compared using two-way ANOVA with Šidák’s post-test, and statistical significance is indicated by * *p* < 0.05, # *p* < 0.0001.

Given that altered Mip activity influences pathogenicity in infection with human macrophages (Figure 2D), we hypothesized that Mip activity may influence metabolism in infections. To better understand how Mip influences *Lp* metabolism, we applied our metabolic workflow using mass spectrometry approaches (Figure 1). To identify potential metabolic vulnerabilities that may influence *Lp* virulence, we characterized the metabolism in genetically engineered *Lp* strains lacking Mip activity [14] and compared it to that of the wild-type strain (wt). We robustly quantified intracellular metabolites and observed changes in amino acid metabolism, as well as metabolites associated with central carbon metabolism, including TCA cycle metabolism (Figure 3A).

We observed that Mip activity significantly impacts *Lp* metabolism, indicating a role of this virulence factor in reprogramming bacterial metabolism (Figure 3A). Notably, amino acid metabolism was highly affected in Δ*mip* strains, suggesting a potential impact on nitrogen homeostasis. Specifically, Δ*mip* strains exhibited significantly increased levels of the BCAAs leucine, isoleucine, and valine, indicating that Mip influences BCAA metabolism in *Lp* (Figure 3B). Notably, many metabolites associated with TCA cycle metabolism, including citrate, were not affected (Figure 3C). The lack of Mip activity significantly increased malate levels, further supporting that Mip induces reprogramming of specific metabolic pathways rather than affecting overall global metabolism (Figure 3C). Thus, our metabolic study identified that Mip activity impacts bacterial metabolism, linking virulence factors to metabolic alterations in *Lp*.

### 3.3. Metabolic Impact of Chemical Inhibition of PPIase in Lp

Our metabolic studies using genetically engineered *Lp* strains provide evidence that Mip activity has a significant impact on amino acid metabolism, particularly BCAAs (Figure 3). To further support our observation, we applied the PPIase inhibitors FK506 and rapamycin to modify Mip activity. Despite the well-known immunosuppressive properties of FK506 and rapamycin [22,23], their effects on *Lp* metabolism have not been well characterized.

**Figure 4:**
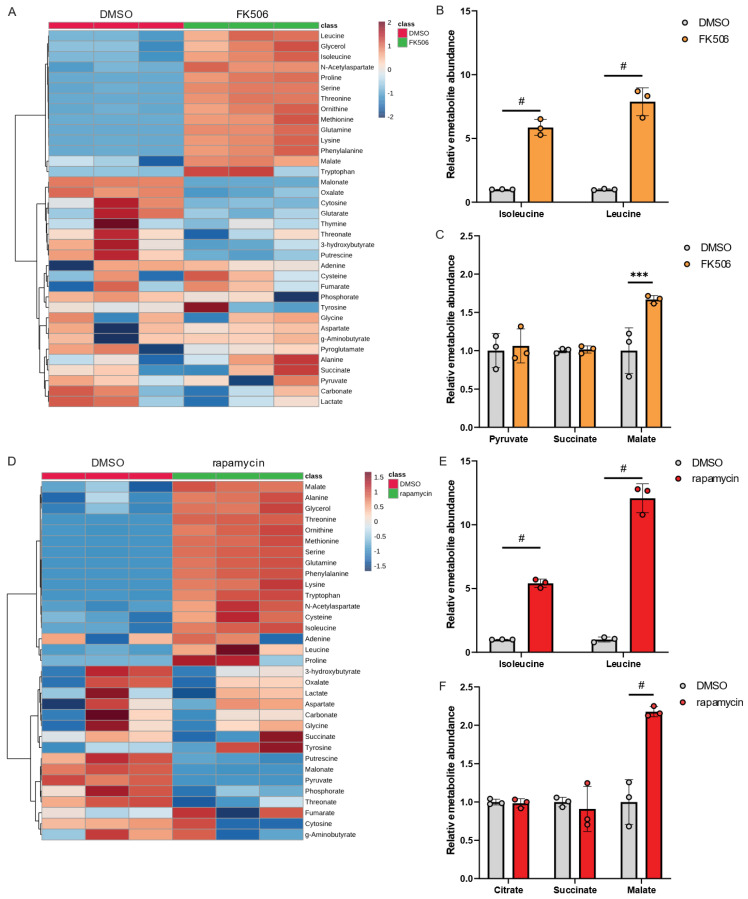
FK506 and rapamycin influence BCAA metabolism in *Lp*. (**A**) Heatmap depicting metabolic changes in response to FK506 treatment. (**B**) Relative metabolite abundance of BCAAs in response to FK506 treatment. (**C**) Relative metabolite abundance of TCA cycle metabolites in response to FK506 treatment. (**D**) Heatmap depicting abundances of metabolites in response to rapamycin treatment. (**E**) Relative metabolite abundance of BCAAs in response to rapamycin treatment. (**F**) Relative metabolite abundance of TCA cycle metabolites in response to rapamycin treatment. *Lp* wild-type strains were exposed to 20 µM FK506 or 20 µM rapamycin and compared to DMSO control. All data are presented as mean ± s.e.m. calculated from n = 3 cultural replicates, and each experiment was repeated independently three times. Relative abundances were compared using two-way ANOVA with Šidák’s post-test, and statistical significance is indicated by *** *p* < 0.001, # *p* < 0.0001.

To investigate the impact of chemical inhibition of PPIase activity in *Lp*, we exposed *Lp* to FK506 and quantified metabolic changes compared to control conditions using our metabolic workflow outlined in Figure 1. Our findings revealed that FK506 treatment alters *Lp* metabolism, with the most significant effects observed in amino acid metabolism (Figure 4A). Notably, FK506 treatment increased levels of BCAAs (Figure 4B), while TCA cycle metabolism remained largely unaffected (Figure 4C). We further examined the effects of the PPIase inhibitor rapamycin on *Lp* metabolism and observed metabolic reprogramming in response to rapamycin (Figure 4D). Similar to FK506 treatments, we observed significant increases in BCAA levels (Figure 4E) and minor changes in TCA cycle metabolism (Figure 4F), further indicating that the virulence factor Mip influences amino acid levels in *Lp*.

Collectively, chemical inhibition of PPIase using FK506 or rapamycin recapitulates the metabolic reprogramming observed in genetically engineered Δ*mip* strains. Our findings provide evidence for the involvement of PPIase activity in modulating *Lp* metabolism, particularly BCAA metabolism, which may affect the virulence of this organism.

### 3.4. Metabolic Consequences of Altered PPIase Activity in Human Macrophages

Our data depicted in Figure 3 and Figure 4 revealed that the virulence factor Mip modulates *Lp* metabolism. Although *Lp* induces host cellular responses upon infecting macrophages, the effects of Mip on macrophage metabolism have largely remained unknown. Previous research demonstrated the structural and functional homology between bacterial Mip and mammalian FK506-binding protein (FKBP) [24]. Notably, rapamycin has immunoregulatory functions and inhibits the PPIase activity of human FKBP12 [25]. To investigate the potential role of Mip in pathometabolism, we used the human leukemia monocytic cell line THP-1 as a host model system and differentiated cells into macrophages. We then analyzed metabolic changes in response to rapamycin, which influences the PPIase activity of FKBP in human macrophages, and we compared the results to our findings obtained from *Lp* (Figure 4).

**Figure 5:**
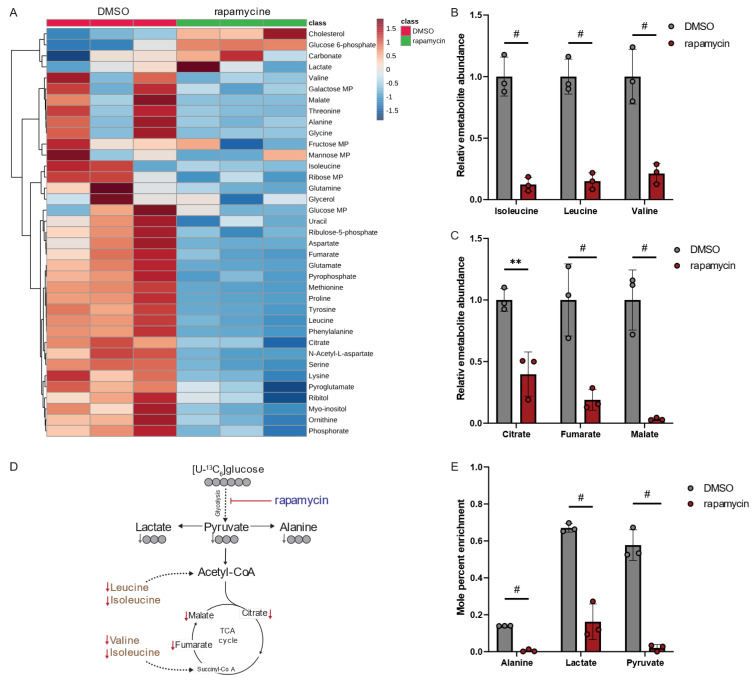
Rapamycin alters BCAA and TCA cycle metabolism in macrophage-like THP-1 cells. (**A**) Heatmap depicting metabolic changes in response to rapamycin. (**B**) Relative metabolite abundance of BCAAs in response to rapamycin treatment. (**C**) Relative metabolite abundance of TCA cycle metabolites in response to rapamycin treatment. (**D**) Schematic depicting metabolite abundances and carbon distribution on metabolites upon cultivation with [U^13^-C_6_]glucose tracer. Red arrows indicate decreased metabolite levels, and gray arrows indicate decreased labeling on metabolites. (**E**) Mole percent enrichment (MPE) of ^13^C-carbon atoms from [U^13^-C_6_]glucose tracer incorporated into metabolites in response to rapamycin treatment. Cells were treated with 20 µM rapamycin and compared to DMSO controls. All data are presented as mean ± s.e.m. calculated from n = 3 cellular replicates, and each experiment was repeated independently three times. Relative abundances were compared using two-way ANOVA with Šidák’s post-test, and statistical significance is indicated by ** *p* < 0.01, # *p* < 0.0001.

Our metabolomics study demonstrated that rapamycin led to alterations in human macrophage metabolism (Figure 5A). Interestingly, rapamycin significantly decreased intracellular levels of the BCAAs leucine, isoleucine, and valine (Figure 5B), as well as metabolites associated with the TCA cycle metabolism (Figure 5C). These findings suggest that rapamycin has a profound effect on the central carbon metabolism of mammalian hosts. Notably, the metabolic signature in macrophages is distinct from the metabolic reprogramming observed in *Lp* when exposed to rapamycin (Figure 5D,E). Our results demonstrate that, while rapamycin treatments increased BCAA levels in *Lp* bacteria (Figure 4E), it led to a significant decrease in BCAA levels in immune cells (Figure 5B).

Rapamycin also inhibits the mammalian target of rapamycin (mTOR) signaling pathway in mammalian cells influencing cell metabolism and function [26]. Specifically, mTOR activity induces glucose metabolism, which is impaired following treatment with rapamycin [27]. To further decipher the metabolic changes, we applied isotopic tracing approaches and cultured macrophage-like THP-1 cells in media supplemented with [U^13^-C_6_]glucose in the presence of rapamycin (Figure 5D). Rapamycin significantly decreased glycolytic fluxes (Figure 5E), confirming the impact of rapamycin in regulating metabolic fluxes [28]. Overall, our findings suggest that treatment with the PPIase inhibitor rapamycin induces distinct metabolic signatures in the host compared to the pathogen, which may have implications for metabolic cross-talk during infections. Thus, the virulence factor Mip may influence the pathogenicity of *Lp* by alternating the metabolism of the pathogen. Moreover, PPIase inhibitors influence host cell metabolism, which may change susceptibility to infection.

## 4. Discussion

In our study, we investigated the modulatory effects of the virulence factor Mip in the pathometabolism of *Lp*. Given the importance of metabolism in driving cell functions, we hypothesized that Mip activity plays a crucial role in inducing metabolic changes during *Lp* infections. Using our established metabolic workflow, we demonstrated that genetically altered Mip activity or chemical inhibition of PPIase activity induced a characteristic metabolic response in *Lp*. Specifically, we observed that impaired PPIase activity in *Lp* led to a significant increase in BCAA levels, linking Mip activity to metabolic changes that may influence infection. Notably, we found that metabolic alterations in human macrophages in response to rapamycin were distinct compared to *Lp* metabolism, suggesting that PPIase activity may play a role in host–pathogen interactions. Overall, our study suggests that Mip activity may alter the pathometabolism to modulate infection [10].

The growth of *Lp* is dependent on certain amino acids, including BCAAs, cysteine, threonine, and serine. We found that a lack of Mip activity increased the levels of these amino acids in *Lp*, particularly BCAAs, as observed in Δ*mip* strains (Figure 3) and in treatment with chemical PPIase inhibitors FK506 and rapamycin (Figure 4). These data suggest that alterations in amino acid metabolism may be beneficial for the pathogenicity of *Lp*. Our findings also indicated that the loss of Mip activity decreases pathogenicity, suggesting that metabolic reprogramming may influence cell function and survival (Figure 2 and Figure 3). The metabolic compensation in *Lp* may further affect host–pathogen interactions and metabolic homeostasis in host macrophages upon infection. Therefore, BCAA metabolism may represent a metabolic vulnerability that could be targeted therapeutically to alter the outcome of LD.

*Lp* infections induce a complex metabolic and cellular metabolic response in the host and the pathogen. Notably, mTOR represents a promising target that can influence host–pathogen interactions. Reduced mTOR activity likely enhances host defenses against pathogens, while increased activation can benefit pathogen expansion [29]. BCAAs are known to stimulate mTOR while immunosuppressive drugs, such as rapamycin inhibit mTOR signaling [30,31]. Our study revealed that Mip influences BCAA homeostasis, potentially modulating mTOR activity in host cells. Thus, GC/MS-based metabolomics facilitates novel insights into the metabolic patterns underlying LD, albeit with some limitations in terms of chemical coverage. Future studies can include liquid chromatogram (LC)/MS approaches to gain further insights into a broader spectrum of metabolites and pathways. Further studies may also incorporate tracing approaches and co-culture model systems to better understand how virulence factors alter metabolism, which may impact host–pathogen interactions. Mass spectrometry-based workflows and tracing approaches can provide further insight into complex metabolic reprogramming in LD [13].

As we continue to unravel the complexities of the cellular mechanisms involved in *Lp* infection, the importance of metabolism becomes increasingly evident. Our study demonstrated metabolic signatures in *Lp* that are influenced by the activity of the virulence factor Mip, and it provides an important step toward a better understanding of the pathometabolism during *Lp* infections.

## Data Availability

Source data for all figures have been deposited in the repository platform of Technische Universität Braunschweig. The data presented in this study are openly available at doi: https://doi.org/10.24355/dbbs.084-202307051342-0 (accessed on 2 July 2023).

## Supplementary Materials

The following supporting information can be downloaded at: https://www.mdpi.com/article/10.3390/metabo13070834/s1, Figure S1: Raw data for *Lp* growth curves in response to altered MIP activity. Growth curve of *Lp* (**A**) wild-type strain and (**B**) Δ*mip* strain. Data depict growth obtained from n = 6 replicates.

## Abbreviations

^12^C	12-carbon
^13^C	13-carbon
*Δmip* strain	*Mip* knockout strain
ACES	N-(2-acetamido)-2-aminoethanesulfonic acid
BCAAs	Branched-chain amino acids
BCYE	Buffered charcoal yeast extract
BP	By-product
Cfu	Colony forming unit
DMSO	Dimethyl sulfoxide
ECM	Extracellular matrix
FKBP	FK506-binding protein
GC/MS	Gas chromatograph coupled to mass spectrometry
LC/MS	Liquid chromatograph coupled to mass spectrometry
LD	Legionnaires’ disease
*Lp*	*Legionella pneumophila*
Mip	Macrophage infectivity potentiator protein
MP	Main product
MSTFA	N-methyl-N-(trimethylsilyl)trifluoracetamid
MTBSTFA	N-(tert-butyldimethylsilyl)-N-methyltrifluoracetamid
MOI	Multiplicity of infection
mTOR	Mammalian target of rapamycin
PMA	Phorbol 12-myristate 13-acetate
PPIase	Peptidyl-prolyl-*cis/trans*-isomerase
Wt	Wild type
YEB	Yeast extract beef
