# Supplementary material for: The Virulence Factor Macrophage Infectivity Potentiator (Mip) Influences Branched-Chain Amino Acid Metabolism and Pathogenicity of Legionella pneumophila
Source: Metabolites. 2023 Jul 11;13(7):834. doi: 10.3390/metabo13070834 (PMC10386555; doi:10.3390/metabo13070834)
Supplement: Supplementary file 1 [file metabolites-13-00834-s001.zip › metabolites-2398170-supplementary.pdf]

The Virulence Factor Macrophage Infectivity Potentiator (Mip) Influences Branched-Chain Amino Acid Metabolism and Pathogenicity of *Legionella pneumophila*

Fabian Nikolka, Mustafa Safa Karagöz, Mohamed Zakaria Nassef, Karsten Hiller, Michael Steinert and Thekla Cordes

Supplementary Figure S1:

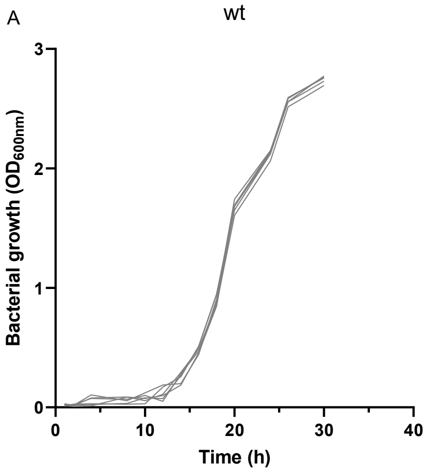

time in h	wt					
X	A:Y1	A:Y2	A:Y3	A:Y4	A:Y5	A:Y6
1	0.0206734	0.0288700	0.0257080	0.0174326	0.0238750	0.0106220
2	0.0147560	0.0163760	0.0126720	0.0073480	0.0212780	0.0255850
4	0.1044080	0.0226250	0.0791530	0.0114020	0.0746270	0.0290100
8	0.0545090	0.0328160	0.0864960	0.0871491	0.0671400	0.0256960
10	0.1216100	0.0803940	0.0742690	0.0548430	0.0989590	0.0294160
12	0.1859340	0.0718890	0.0978650	0.1094300	0.0507770	0.1695840
14	0.1996610	0.2667540	0.1870830	0.2928840	0.2814000	0.2564500
16	0.4427580	0.5110250	0.4566690	0.4898540	0.4716160	0.5100750
18	0.9041230	0.9495140	0.8478560	0.8630470	0.8850010	0.9445440
20	1.7416580	1.6822570	1.6847830	1.6067730	1.6942940	1.6514090
24	2.1497320	2.1342440	2.1222410	2.0614750	2.1442180	2.1211580
26	2.5578050	2.5862300	2.5596980	2.5161760	2.5941410	2.5909060
28	2.6441980	2.6763090	2.6670410	2.6054490	2.6756320	2.6752740
30	2.7305910	2.7663880	2.7743840	2.6947220	2.7571240	2.7596410

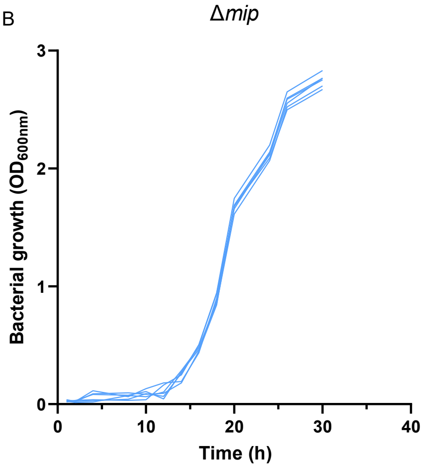

time in h	Δ*mip*					
X	A:Y1	A:Y2	A:Y3	A:Y4	A:Y5	A:Y6
1	0.029517	0.037713	0.034551	0.025011	0.031453	0.018200
2	0.023599	0.025219	0.021515	0.014926	0.028856	0.033163
4	0.113251	0.031468	0.087996	0.018980	0.082205	0.036588
8	0.063352	0.041659	0.095339	0.072249	0.074718	0.033274
10	0.130453	0.089237	0.083112	0.062421	0.106537	0.036994
12	0.179893	0.065848	0.091824	0.102124	0.043471	0.162278
14	0.193620	0.260713	0.181042	0.285578	0.274094	0.249144
16	0.436717	0.504984	0.450628	0.482548	0.464310	0.502769
18	0.898082	0.943473	0.841815	0.855741	0.877695	0.937238
20	1.680923	1.746222	1.678385	1.613047	1.688221	1.657683
24	2.088997	2.198209	2.115843	2.067749	2.138145	2.127432
26	2.497070	2.650195	2.553300	2.522450	2.588068	2.597180
28	2.583463	2.740274	2.660643	2.611723	2.669559	2.681548
30	2.669856	2.830353	2.767986	2.700996	2.751051	2.765915

Figure S1: Figure S1: Raw data for *Lp* growth curves in response to altered MIP activity. Growth curve of *Lp* (**A**) wild-type strain and (**B**) Δ*mip* strain. Data depict growth obtained from n = 6 replicates.
